# *Mycobacterium yongonense* in Pulmonary Disease, Italy

**DOI:** 10.3201/eid1911.130911

**Published:** 2013-11

**Authors:** Enrico Tortoli, Alessandro Mariottini, Piera Pierotti, Tullia M. Simonetti, Gian Maria Rossolini

**Affiliations:** San Raffaele Scientific Institute, Milan, Italy (E. Tortoli);; Careggi University Hospital, Florence, Italy (A. Mariottini, M.T. Simonetti, G.M. Rossolini);; SS. Maria Annunziata Hospital, Florence (P. Pierotti);; University of Florence, Florence (G.M. Rossolini);; University of Siena, Siena, Italy (G.M. Rossolini)

**Keywords:** tuberculosis and other mycobacteria, bacteria, cavitary pulmonary disease, pulmonary disease, respiratory infections, Italy, Mycobacterium yongonense, Mycobacterium avium complex, MAC

**To the Editor:** *Mycobacterium yongonense* is a recently described species (*1*) that belongs to the *M. avium* complex (MAC) and is associated with pulmonary infection. The strain on which the description of species was based was isolated in South Korea from the sputum of a patient with unspecified pulmonary disease. We describe 2 *M. yongonense* strains isolated from patients in Italy. 

Patient 1 was a 74-year-old woman who had experienced fatigue, diarrhea, and weight loss. Her medical history included liver cirrhosis resulting from hepatitis C virus infection and surgery for colon cancer; the patient also reported tuberculosis in childhood. Chest radiograph revealed a cavitary lesion, a finding confirmed by computed tomography scan (Figure). Cultures in liquid and solid media grew a nonchromogenic mycobacterium from sputum and stool samples; results were negative for urine samples.

**Figure:**
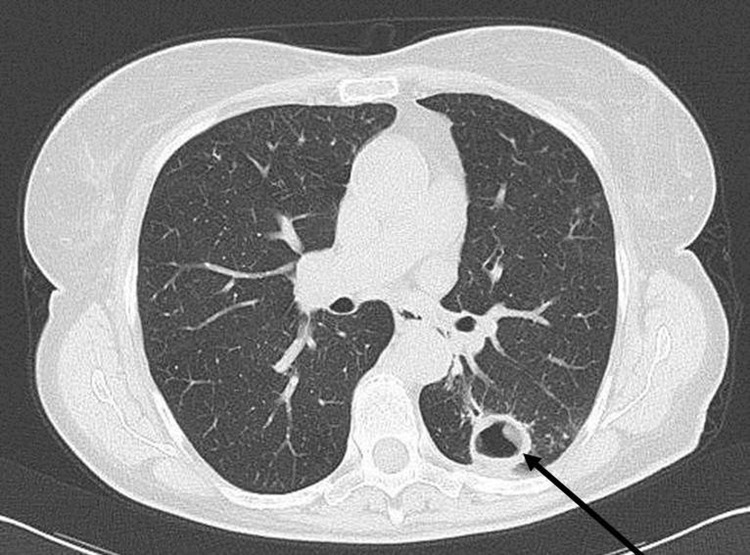
Computed tomography scan showing a cavity (arrow) in the left lung of a 74-year-old woman (patient 1) in Italy. Laboratory testing suggests that the woman was infected with *Mycobacterium yongonense*.

The patient was treated with clarithromycin, rifabutin, and ethambutol and showed some improvement. A bronchoscopic investigation was performed, and microscopic examination of bronchoalveolar lavage samples revealed the presence of acid-fast bacilli that subsequently were grown in culture. The patient began improving markedly starting with the second month of treatment, which will be continued for a total of 18 months.

Patient 2 was a 74-year-old woman, living in a community of nuns, who reported cough and dyspnea. Her medical history included renal failure and surgery for breast cancer. A bronchoalveolar lavage was performed; samples yielded in culture *Pseudomonas aeruginosa* and a nonchromogenic mycobacterium. The patient was treated with cefepime, to which *P. aeruginosa* was susceptible in vitro, and rapidly improved. The isolation of the nontuberculous mycobacterium was considered irrelevant, and no specific treatment was undertaken.

To determine the specific mycobacteria species isolated from these patients, we conducted a commercial line-probe assay (GenoType Mycobacterium CM; Hain Lifesciences, Nehren, Germany). Both strains were identified as *M. intracellulare*. However, the known cross-reaction of *M. intracellulare* probe with most MAC species (*2*) led us to determine the complete sequence of the 16S rRNA gene. Both strains showed 100% similarity with *M. yongonense* and *M. marseillense* (*3*) strains. 

To confirm this unusual finding, we investigated other genetic regions. We detected 100% identity with *M. yongonense* in the internal transcribed spacer 1 region and in a 1,384-bp region of the *hsp65* gene and found 2 mismatches in a 420-bp fragment of the *sodA* gene (99.5% similarity). In contrast, *M. marseillense* showed 6 mismatches (98.6% similarity) in the internal transcribed spacer 1 region and 24 (98.3% similarity) in *hsp65*; no *sodA* sequence is available in GenBank for this species. Partial sequencing of other genetic targets not available in GenBank for *M. yongonense* enabled us to confirm the close relatedness of the strains to *M. intracellulare* (100% similarity in *dnaK* gene; 99.3% identity in *gyrB* and *gyrC* genes).

The finding of the same novel *Mycobacterium* species in these 2 unrelated patients reflects variability in the significance of nontuberculous mycobacteria isolated from clinical specimens. *M. yongonense* was probably a contaminant in the second case, but in the first, its involvement as causative agent of disease seems incontrovertible. The specific criteria of the American Thoracic Society (*4*) were fulfilled: radiographic imaging clearly documented the presence of a cavitary pulmonary lesion, no other pathogen possibly responsible of disease was detected by bronchoscopic investigation, and the same mycobacterium was isolated repeatedly from sputum (its presence in stool probably results from swallowed sputum) and bronchoalveolar lavage samples. Confirmation is further provided by the response to the specific therapy, according to international guidelines (*4**,**5*), for MAC pulmonary disease (MICs were 2, 1, and 8 µg/mL for clarithromycin, rifabutin, and ethambutol, respectively).

The initial description of *M. yongonense* noted that it has a distinct *rpoB* sequence (*1*), identical to that of a distantly related scotochromogenic species, *M. parascrofulaceum*. In a more recent article (*6*), the same authors investigated 2 more strains of *M. yongonense* with similar characteristics and suggested that the recent acquisition of the *rpoB* gene resulted from a lateral gene transfer event from *M. parascrofulaceum*. The *rpoB* genes of the strains we investigated, however, were substantially different from that of *M. scrofulaceum* and were instead related to that of *M. intracellulare* (99.4% similarity) and, less closely, to that of other species belonging to the MAC, including *M. marseillense* (97.4%). Discrepancy in the *rpoB* sequence means some uncertainty remains that our strains are *M. yongonense*, but the 100% identity in major phylogenetically relevant regions strongly supports this hypothesis and suggests the possibility of a variant of the species preceding the acquisition of the *rpoB* gene from *M. parascrofulaceum*. Less evidence exists for identifying the strains as *M. marseillense* because of the clear divergence in the genes investigated, other than 16S rRNA. 

The complete epidemiology of *M. youngonense* is unknown, in part because few strains have been identified. However, as in the cases we describe, use of suboptimal identification methods may mean that some isolates have been misidentified as other mycobacteria species.
